# A recombinant bovine herpesvirus-4 vectored vaccine delivered via intranasal nebulization elicits viral neutralizing antibody titers in cattle

**DOI:** 10.1371/journal.pone.0215605

**Published:** 2019-04-19

**Authors:** Laura B. A. Williams, Lindsay M. Fry, David R. Herndon, Valentina Franceschi, David A. Schneider, Gaetano Donofrio, Donald P. Knowles

**Affiliations:** 1 Department of Veterinary Microbiology & Pathology, Washington State University, Pullman, Washington, United States of America; 2 Animal Disease Research Unit, Agricultural Research Service, United States Department of Agriculture, Pullman, Washington, United States of America; 3 Department of Medical-Veterinary Science, University of Parma, Parma, Italy; Instituto Butantan, BRAZIL

## Abstract

Recombinant herpesvirus vaccine vectors offer distinct advantages in next-generation vaccine development, primarily due to the ability to establish persistent infections to provide sustainable antigen responses in the host. Recombinant bovine herpesvirus-4 (BoHV-4) has been previously shown to elicit protective immunity in model laboratory animal species against a variety of pathogens. For the first time, we describe the induction of antigen-specific immune responses to two delivered antigens in the host species after intranasal nebulization of recombinant BoHV-4 expressing the chimeric peptide containing the bovine viral diarrhea virus (BVDV) glycoprotein E2 and the bovine herpesvirus 1 (BoHV-1) glycoprotein D (BoHV-4-A-CMV-IgK-gE2gD-TM). In this study, four cattle were immunized via intranasal nebulization with the recombinant BoHV-4 construct. Two of the cattle were previously infected with wild-type BoHV-4, and both developed detectable serologic responses to BVDV and BoHV-1. All four immunized cattle developed detectable viral neutralizing antibody responses to BVDV, and one steer developed a transient viral neutralizing response to BoHV-1. Approximately one year after immunization, immunosuppressive doses of the glucocorticoid dexamethasone were administered intravenously to all four cattle. Within two weeks of immunosuppression, all animals developed viral neutralizing antibody responses to BoHV-1, and all animals maintained BVDV viral neutralizing capacity. Overall, nebulization of BoHV-4-A-CMV-IgK-gE2gD-TM persistently infects cattle, is capable of eliciting antigen-specific immunity following immunization, including in the presence of pre-existing BoHV-4 immunity, and recrudescence of the virus boosts the immune response to BoHV-4-vectored antigens. These results indicate that BoHV-4 is a viable and attractive vaccine delivery platform for use in cattle.

## Introduction

Bovine herpesvirus-4 (BoHV-4) is within the rhadinovirus genus in the gammaherpesvirus subfamily of the Herpesviridae family [1]. As is typical of other gammaherpesviruses, BoHV-4 initially infects mucosal epithelial cells, and replicates and undergoes latency in monocytes and macrophages [2]. BoHV-4 has been isolated from a variety of tissues from healthy cattle [3, 4]. Although BoHV-4 has also been identified in diseased respiratory, enteric, and most often, reproductive tissues, causal associations with disease have not been definitively established, and experimental infection of cattle does not reliably result in overt clinical disease [5, 6].

Herpesviruses have intrinsic properties that make them attractive for use as viral vaccine vector candidates. Herpesviruses are capable of incorporating large amounts of foreign genetic material and can undergo replication-competent persistent infections in the host. For example, herpesvirus of turkeys (HVT) has been developed as successful, single dose veterinary vaccine capable of producing long term protective responses against avian influenza in chickens based in part on the ability to undergo persistent infection, even in the presence of neutralizing maternal antibodies [7–10]. Thus herpesvirus vectors are thought to be capable of priming and maintaining lifelong antigen-specific protective immunity. In addition to these intrinsic biological properties of herpesviruses, BoHV-4 is non-oncogenic and avirulent under experimental challenge conditions [6]. In light of these properties, BoHV-4 has been successfully developed as a veterinary vaccine vector in model animal species against a variety of infectious diseases. BoHV-4-vectored vaccines have successfully induced neutralizing antibody responses to bovine herpesvirus-1 (BoHV-1) and Bovine Viral Diarrhea Virus (BVDV) type 1 in rabbits [11, 12], neutralizing antibody responses to BVDV type 1 in sheep [13], neutralizing antibody responses to Bluetongue virus and to Peste de Petits Ruminants (PPR) virus in mice, respectively [14, 15], and protective viral neutralizing antibody responses against caprine herpesvirus-1 in goats [16]. Despite the success of the BoHV-4 platform in these non-BoHV-4 host species, and the intrinsic benefits of the BoHV-4 platform, immunization using BoHV-4 has never been attempted in cattle. The seroprevalence of BoHV-4 in cattle populations varies widely in different geographical regions; recently, typical global seroprevalence estimates vary between 4–30% [17]. Some dairy herds experience up to 88% seroprevalence [18] and wild African buffalo populations reportedly have up to 94% seroprevalence [19]. Given the seroprevalence of BoHV-4 in most bovid populations, it is critical to determine whether pre-existing immunity directed at the vector could preclude the development of antibody responses to the vaccine antigen after BoHV-4-vectored immunization. This is a concern with other viral vaccine delivery systems, particularly with the use of adenoviral vectors [20]. Specifically, reduction in recombinant adenoviral vaccine vector efficiency attributed to decreased transgene transduction has been described following vaccination of humans that had pre-existing neutralizing antibodies developed to circulating strains of adenovirus [21].

The immunodominant BVDV type 1-derived antigen E2 and BoHV-1-derived glycoprotein D were used as the model immunogens, and were expressed as a chimeric protein by recombinant BoHV-4 (BoHV-4-A-CMV-IgK-gE2gD-TM). This recombinant BoHV-4 construct expressing E2 and gD has been used to effectively produce antigen-specific immunity in previous studies using a rabbit model [12, 22]. Additionally, E2 and gD antigens have been experimentally shown to incite protective immune responses in cattle to BVDV and BoHV-1, respectively [23, 24]. The VN immune response reported in these experimental subunit vaccines meets the standardized requirements for commercial vaccines that protect against BVDV and BoHV-1 associated disease according to the United States Department of Agriculture Animal Plant Health inspection services Code of Federal Regulations (9 CFR § 113.215 and 113.216, respectively) [25]. The development of VN antibody titers correlates with clinical immune protection from disease, and thereby abrogates the requirement to challenge immunized cattle with virulent virus to verify protection [26]. The use of model antigens that obviate challenge studies are desirable to meet Assessment and Accreditation of Laboratory Animal Care (AALAC) and Animal Welfare Act goals of replacement, reduction, and refinement of animal use in research.

The aims of this study were three-fold: to assess whether immunization of cattle with recombinant BoHV-4 expressing model antigens could elicit antigen-specific protective immune responses, to determine whether recombinant BoHV-4 can persistently infect cattle, and to determine whether pre-existing immunity to BoHV-4 precludes its use as a vaccine vector in cattle. The results of this study provide evidence of the utility of recombinant BoHV-4 as a vaccine platform for cattle, and the benefits of this vaccine platform are discussed in light of our findings.

## Materials and methods

### Virus generation

The wild-type and recombinant BoHV-4 viruses used in this study were constructed as previously described [22]. Briefly, the BoHV-4 genome was originally cloned as a bacterial artificial chromosome, and the BAC cassette was inserted into the BoHV-4 genome by homologous recombination (pBAC-BoHV-4-A). The thymidine kinase (TK) region was targeted by insertion of a galactokinase prokaryotic expression cassette (GalK) and kanamycin resistance expression cassette (KanaGalK) flanked by the TK region via electroporation and subsequent double positive selection (pTK-KanaGalK-TK). The pCMV-IgK-gE2gD-TM plasmid flanked by the TK region retargeted the selection cassette during heat-inducible homologous recombination to produce pBAC-BoHV-4-A-CMV-IgK-gE2gD-TM. Transfected cell membrane-associated protein expression was detected by both anti-gD and gE2 monoclonal antibodies by Western immunoblotting as previously described [22]. Electroporation into BEKcre cells expressing cre recombinase eliminates the BAC cassette from the viral genome. Infectious BoHV-4-A-CMV-IgK-gE2gD-TM virus is obtained by freezing and thawing BEKcre cells three times and pelleting virions through 30% sucrose. WT BoHV-4 is generated similarly by electroporating pBAC-BoHV-4-A into BEKcre cells expressing cre recombinase, undergoing three freeze/thaw cycles, and pelleting the virions. Viruses were propagated by infecting confluent monolayers of Madin Darby Bovine Kidney (MDBK) cells at an MOI of 0.5 TCID_50_ per cell and maintaining them in minimal essential medium (MEM) with 10% fetal bovine serum (FBS) for two hours. The medium was then removed and replaced with fresh MEM containing 10% FBS. When approximately 90% of the cell monolayer exhibited cytopathic effect (CPE), approximately 72 hours postinfection, the virus was prepared by freezing and thawing cells three times and pelleting virions through 30% sucrose. Virus pellets were resuspended in cold MEM without FBS. TCID_50_ were determined on MDBK.

MDBK cells were infected at an MOI of 0.5 PFU/cell and incubated at 37°C for four hours. Infected cells were washed with serum-free Eagle’s MEM and then overlaid with Eagle’s MEM containing 10% FBS, 2 mM of l-glutamine, 100 IU/ml of penicillin (Sigma-Aldrich, St. Louis, Missouri, USA), 100 μg/ml of streptomycin (Sigma-Aldrich) and 2.5 μg/ml of amphotericin B. The supernatants of infected cultures were harvested every 24 h, and the amount of infectious virus was determined.

### Cattle

Four, six-month-old Holstein-cross steers were acquired from a dairy in central Washington and maintained in accordance with the Washington State University and University of Idaho Institutional Animal Care and Use Committees, approved protocol numbers 04596 (WSU), 04975 (WSU) and 2016–18 (U of I). The four cattle were housed together at approved WSU and USDA-ARS research barns in Moscow, ID and Pullman, WA for the duration of this study. Cattle were quarantined for two weeks following acquisition, and were serologically tested for the presence of pre-existing antibodies to BoHV-1, BoHV-4, and BVDV during this time.

### Infection of cattle with WT BoHV-4

Two of the steers (steers 1 and 2) were infected with 2.0 mL of a 10^7^ TCID_50_ /mL preparation of WT BoHV-4 via nebulization, as previously described [27]. Briefly, cattle were restrained in a head catch with a large cloth draped over both eyes. Intranasal immunization was achieved by slight modification of a commercially available human nebulization system (Devilbiss PulmoAide nebulizer, Sunrise Medical, Somerset, PA). One end of a 20 cm long, 0.5 cm diameter, PVC polymer clear plastic tube was fitted to the chamber of the nebulizer, and the other end was inserted approximately 5 cm into the left nasal passage of each animal. Over a six minute period, the 2 mL virus preparation was delivered via nebulization as the steers respired. A physical examination, including assessment of rectal temperature, pulse, and respiration rate, was performed daily for 14 days post-infection. Blood was collected via jugular venipuncture every other day for three weeks and then weekly for the remainder of the study for use in serologic, molecular, and viral-culture based assays.

### Immunization of cattle

All steers were immunized with recombinant BoHV-4-A-CMV-IgK-gE2gD-TM. Steers 1 and 2, which were first infected with WT BoHV-4 were immunized with 2.0 mL of a 10^5^ TCID_50_ /mL preparation of BoHV-4-A-CMV-IgK-gE2gD-TM via nebulization as described above approximately six months after infection with WT BoHV-4. At this time, the remaining two age-matched steers with undetectable BoHV-4 antibody titers (steers 3 and 4) were immunized with an identical preparation of BoHV-4-A-CMV-IgK-gE2gD-TM. All cattle were boosted with the same recombinant viral preparation via the intranasal nebulization route ten weeks later. Blood collection and physical examinations were performed as described for WT BoHV-4 infection.

### Dexamethasone suppression

Dexamethasone suppression was performed as described previously [28]. Briefly, 0.1 mg/kg dexamethasone was administered intravenously to each steer once daily for five days. For the next 14 days, cattle were examined daily for evidence of recrudescent herpesviral infection, including elevated rectal temperature, nasal or ocular discharge, mucosal lesions, cough, and elevated heart or respiratory rate. Ten days prior to euthanasia, dexamethasone suppression was repeated via administration of 0.1 mg/kg dexamethasone intravenously to each steer once daily for three days. Blood was collected at regular intervals via jugular venipuncture following dexamethasone suppression. Nasal swabs were collected every other day for two weeks following dexamethasone administration. Nasal specimens were collected bilaterally from the nares of each steer with a dry swab by inserting the tip of the collection swab approximately 5 cm into the nares and rolled five times, and were transported in universal transport medium (BD Diagnostics, Sparks, MD, USA). Collected specimens were transported at room temperature and stored at -20°C.

### Post-mortem analysis

Cattle were euthanized via intravenous injection of sodium pentobarbital (Fatal Plus; Vortech Pharmaceuticals, USA) and a complete necropsy performed on all steers within two hours of death. Fresh samples of the trigeminal nerve ganglia, brainstem, cerebral cortex, lung, liver, kidney, spleen, tracheobronchial lymph node, and bone marrow were collected and frozen at -20°C for use in viral isolation.

### BoHV-4 viral isolation

All BoHV-4 isolations were performed using MDBK cells in Dulbecco’s Modified Eagle’s Medium (DMEM) media (Sigma-Aldrich) supplemented with 10% FBS (Sigma-Aldrich), 2 mM l-glutamine (Sigma-Aldrich), 100 IU/ml of penicillin (Sigma-Aldrich), and 100 μg/ml of streptomycin (Sigma-Aldrich), referred to as complete DMEM (cDMEM). Co-cultures were performed from the peripheral blood leukocyte fraction (PBL; buffy coat) of approximately 10 mL of whole blood collected into EDTA Vacutainer tubes (BD Diagnostics) from steers. Blood was centrifuged at 1000 x g for 15 minutes, and the buffy coat transferred to a new 15 mL conical plastic tube. The cells were washed three times in PBS, and resuspended in 1.0 mL cDMEM. 200 uL of this preparation was inoculated into duplicate wells of a 24-well plate containing 90% confluent MDBK cells and cultured at 37°C supplemented with 5% CO_2_ in cDMEM. Cells were visualized daily using an inverted microscope for the appearance of CPE. After five days, MDBKs that had not developed CPE were passaged using three, -80°C freeze/thaw cycles followed by centrifugation at 1300 x g for 5 minutes to remove cellular debris. The supernatant was inoculated onto fresh MDBKs for observation. Cells that developed CPE were prepared for DNA extraction and PCR analysis to confirm BoHV-4 infection. Viral isolation from nasal swab media was performed similarly using transport media that has undergone three -80°C freeze/thaw cycles and subsequent inoculation of 200 uL of the transport media onto 90% confluent MDBK cells as described above. For isolation from tissue samples, tissues collected postmortem were minced into 0.1 to 0.5 cm^3^ segments and suspended in 0.25% trypsin at 37°C for five minutes. The samples were centrifuged at 150 g for 10 min, and the supernatant mixed 1:1 in cDMEM and overlaid onto 90% confluent MDBK cells in 24-well tissue culture plates at 37°C and 5% CO_2_, as described above.

### DNA extraction and BoHV-4 PCR

Genomic DNA was extracted (DNEasy Blood and Tissue Kit, Qiagen, Maryland, USA) and eluted in a final volume of 100 μL. PCR was performed using Accuprime Pfx Supermix (ThermoFisher, Austin, Texas, USA) in a 27 μL reaction volume using 2 μL DNA template and 450 nM of each primer. The primer sequences were obtained from the inner portion of PCR described previously for use in nested PCR [5, 29]: forward primer sequence (5’-TTGATAGTGCGTTGTTGGGATGTGGT-3’) and reverse primer sequence (5’CACTGCCCGGTGGGAAATAGCA-3’). PCR cycling was 95°C for 5 minutes followed by 35 cycles of 94°C for 30 seconds, 55°C for 10 seconds and 68°C for 30 seconds followed by electrophoresis on a 1.25%, 1x TAE agarose gel. Negative control samples included DNA extracted from uninfected MDBK cells and positive control samples included DNA extracted from MDBKs infected *in vitro* with 1.0 Multiplicity of Infection (MOI) BoHV-4-A.

### BoHV-4 serology

An indirect BoHV-4 ELISA (BIO-X, Brussels, Belgium) was performed per the manufacturer’s instructions. Briefly, microtiter plates were coated in pairs with purified BoHV-4 and negative control cell lysate provided by the manufacturer. Kit positive and negative serum samples and test serum samples were diluted 1:100 and added to individual wells. After washing, each well was incubated in horseradish peroxidase-labeled conjugate, washed, and incubated for ten minutes in the provided chromogen substrate. The color reaction was stopped with phosphoric acid and the optical density (OD) value was determined by an ELISA plate reader at 450 nm. Percent positivity was calculated by dividing the difference in the OD of the cell lysate and virus-coated wells for each sample by the difference in OD of the kit-provided positive sample and multiplied by 100, as shown:

$$Value\;(percent\;positivity)\;=\;\frac{Delta\;OD\;(sample\;wells)}{Delta\;OD\;(positive\;control\;wells)}\;x\;100$$

The positive cut-off suggested by the manufacturer is 30% positivity. The cut-off for positivity routinely used in our lab is 14.5%. This is based on previous serologic evaluation of regional field samples using 42 cattle, in which the average percent positivity was 7.73%, with a standard deviation of 2.25. The cutoff for a positive was calculated as three times the standard deviation plus the average percent positivity.

### BoHV-1 serology

A commercial BoHV-1 indirect ELISA (IDEXX, Hoofddorp, The Netherlands) was performed as described by the manufacturer instructions. Briefly, manufacturer-provided microtiter plates coated in ultrapurified BoHV-1 infected cell lysate were incubated for one hour at 37°C with duplicate samples of the test serum, and the provided negative and positive control sera. All sera samples were diluted 1:100 in dilution buffer and added to individual wells. After washing three times with wash solution, each well was incubated for 30 minutes at 37°C in horseradish peroxidase-labeled conjugate, washed, and incubated for 20 minutes at room temperature in the provided tetramethylbendizine (TMB) chromogen substrate. The color reaction was stopped with sulfuric acid and the OD value was determined by an ELISA plate reader at 450 nm. Percent positivity was calculated by dividing the difference in the OD of each test sample and negative control by the difference in OD of the kit-provided positive and negative sera samples and multiplied by 100, as shown:

$$Negative\;control\;value\;=\;\frac{Delta\;OD\;(negative\;control\;wells)}{2}$$

$$Positive\;control\;value\;=\;\frac{Delta\;OD\;(positive\;control\;wells)}{2}$$

$$Value\;(percent\;positicity)\;=\;\frac{OD\;(sample)-Negative\;control\;value}{Positive\;control\;value-Negative\;control\;value}$$

The positive cut-off was interpreted in accordance with the manufacturer’s guidelines, with any value above 50% considered positive.

### BVDV and BoHV-1 diagnostic assays

Following immunization, whole blood and serum from each steer were submitted weekly to the Washington Animal Disease Diagnostic Laboratory (WADDL, Pullman, WA) for BVDV and BoHV-1 diagnostic tests. Whole blood in EDTA was used for BVDV PCR and BoHV-1 PCR, and serum used for BVDV virus neutralization, BoHV-1 virus neutralization, and BVDV E^rns^ antigen ELISA testing. WADDL is fully accredited by the American Association of Veterinary Laboratory Diagnosticians (AAVLD) and is a member of the National Animal Health Laboratory Network, and all testing procedures have been validated through these channels.

In brief, for the virus neutralization (VN) assays, each test serum sample and positive and negative controls (NVSL) were initially diluted 1:4 using 50 μL of serum in 150 μL of cDMEM in 96-well plates. Serial two-fold dilutions of each sample were then made in duplicate. Twenty-five μL of virus suspension containing 100 TCID_50_ of BVDV or BoHV-1 (BVDV type 1 SINGER strain, BVDV type 2 125 genotype 2 strain, and BoHV-1 Colorado strain, respectively) were added to respective wells. After one hour of incubation at 37°C, 150 μl of a 10^4^ cells/mL bovine turbinate cell suspension was added to each well and the plates were incubated for three days at 37°C in a humidified incubator supplied with 5% CO_2_. Cytopathic effect (CPE) was detected using inverted light microscopy. Neutralizing antibody titers were expressed as the reciprocal (log 2) of the final dilution of serum that completely inhibited viral infectivity.

A commercially available BVDV type 1 and type 2 E^rns^ antigen detection ELISA (IDEXX, Westbrook, Maine, US) was performed per the manufacturer’s instructions. Briefly, manufacturer-provided microtiter plates coated in E^rns^-specific monoclonal antibody and 50 μL manufacturer-provided detector antibody were incubated for one hour at 37°C with duplicate samples of the test serum, and the provided negative and positive control sera. After washing three times with wash solution, each well was incubated for 30 minutes at 37°C in horseradish peroxidase-labeled conjugate, washed, and incubated for 30 minutes at room temperature in the provided TMB chromogen substrate. The color reaction was stopped with sulfuric acid and the OD value was determined by an ELISA plate reader at 450 nm. Calculations were performed by dividing the difference in the OD of each test sample and negative control by the difference in OD of the kit-provided positive and negative sera samples, as shown:

$$Negative\;control\;value\;=\;\frac{Delta\;OD\;(negative\;control\;wells)}{2}$$

$$Positive\;control\;value\;=\;\frac{Delta\;OD\;(positive\;control\;wells)}{2}$$

$$Value\;=\;\frac{OD\;(sample)-Negative\;control\;value}{Positive\;control\;value-Negative\;control\;value}$$

The positive cut-off was interpreted in accordance with the manufacturer’s guidelines, with any value greater than or equal to 0.15 considered positive.

For BoHV-1 detection from the peripheral blood, a real time PCR (RT-PCR) was used. Genomic DNA was extracted (DNEasy Blood and Tissue Kit; Qiagen) and eluted in a final volume of 100 μL. PCR was performed as previously described [30] using 10 μl Multiplex PCR Master Mix (ThermoFisher), 2.0 μl dNTP mix (Bioline, Memphis, Tennessee, USA), 1.25 μl forward primer, 1.25 μl reverse primer, 2.5 μl probe, and 3.0 μl water in a total reaction volume of 20 uL. The primer sequences were used as follows: forward (5'-TGCCCTACAGGTCGTTGATTA-3'), reverse (5'-TCCAGCTGCCTCCTCTGTTT-3’), and probe sequence 5' FAM-CGTGTGCTTCTCGGCAGTCATCA-BHQ-1 3'. The PCR reactions were performed at 95°C for 15 minutes followed by 40 cycles of 94°C for 60 seconds, 55°C for 30 seconds and 72°C for 30 seconds. Water was used as the negative control sample, and the positive control DNA sample was extracted from BoHV-1 infected cells as described above in the VN assay. The values were obtained from the generated curves where the x–axis represents the PCR cycle number and the y-axis represents the relative fluorescence. Samples with detectable signals were reported as positive (data not shown).

For detection of BVDV types 1 and 2 from the peripheral blood, RT- PCR was performed. RNA extractions were performed using MagMax-96 Viral RNA Isolation Kit (ThermoFisher), and eluted in a final volume of 90 μL. Real time PCR was performed as previously described [31] using the BVDV detection kit (Vet-MAX Gold, ThermoFisher, Austin, Texas, USA) containing 12.5μl of RT-PCR buffer, 1.0 μl BVDV primer/probe, 0.2 μl XenoRNA-01, and 1.0 μl RT-PCR enzyme mix in a total reaction volume of 14.7 μL. The primer sequences were used as follows: forward sequence (5’-GTAGTCGTCAGTGGTTCG-3’), and a proprietary reverse primer and probe. The PCR reactions were performed at 45°C for 10 minutes, 95°C for 10 minutes, followed by 40 cycles of 95°C for 15 seconds, 60°C for 45 seconds. Water was used as the negative control sample, and the positive control DNA sample was extracted from BVDV (BVDV Type I Singer strain, NVSL) infected cells as described above in the BVDV VN assay. The control-based threshold (Ct) values were obtained from the generated curves, and samples with Ct values below or equal to 38.0 were reported as positive (data not shown).

## Results

### Infection of cattle with WT BoHV-4 via intranasal nebulization

**Fig 1:**
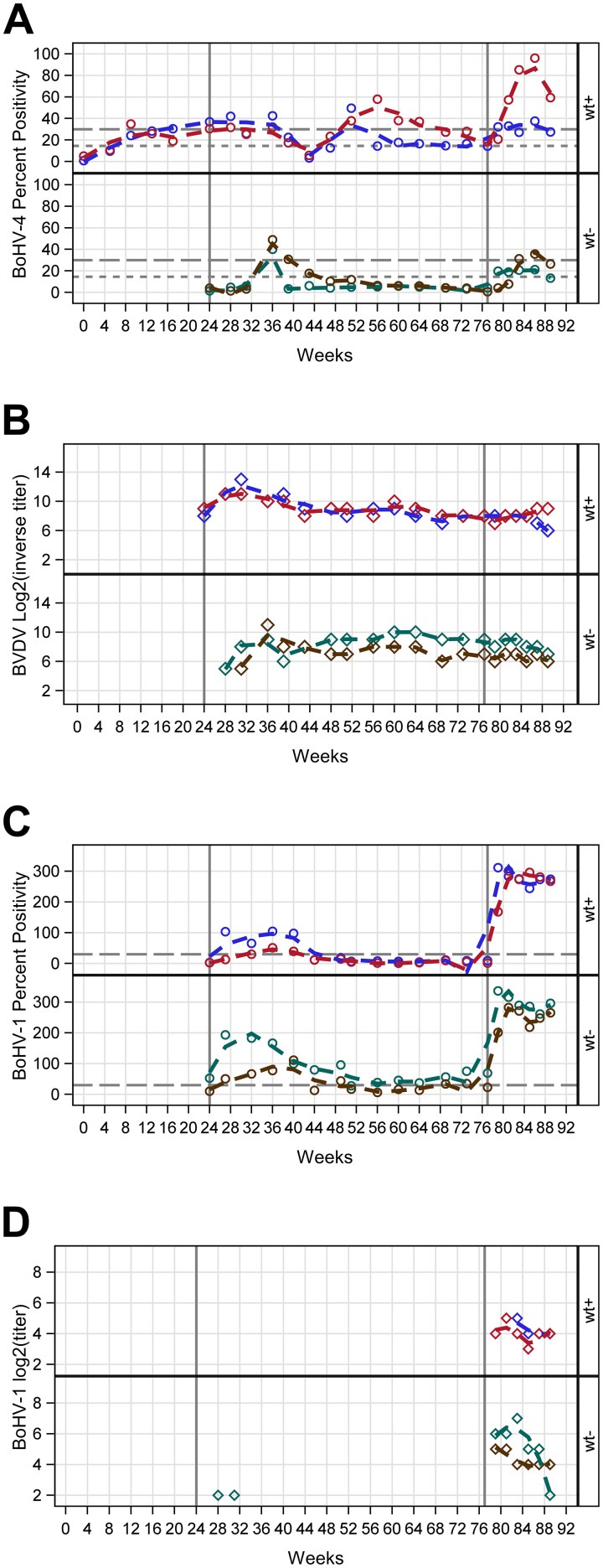
Antibody responses to antigens in each steer. Data trends were fit by Loess (degree = 2, smoothing = 0.3), and original data are shown as symbols. The solid gray lines indicate positive cut-off values as suggested by the kit manufacturer for each respective ELISA kit, and the dotted grey line indicates the positive cut-off value as calculated for the geographical region. Steers 1 (blue) and 2 (red) (top) were WT-BoHV-4- infected (wt +) and steers 3 (green) and 4 (brown) (bottom) were immunized only with BoHV-4-A-CMV-IgK-gE2gD-TM (wt -). WT-BoHV-4 infection is indicated in the first 24 weeks, immunization with BoHV-4-A-CMV-IgK-gE2gD-TM occurred at week 24, and dexamethasone-induced suppression took place during week 77. **Panel A.** BoHV-4 antibody percent positivity as determined by indirect ELISA. **Panel B.** BVDV antibody response as determined by VN. **Panel C.** BoHV-1 antibody response as determined by indirect ELISA. **Panel D.** BoHV-1 antibody response as determined by VN.

We tested whether cattle could be infected with BoHV-4 via the respiratory route using intranasal nebulization. Seven days post-infection (PI), steers 1 and 2 developed a marked, transient pyrexia of 40.6°C and 40.9°C, respectively. On all other days within the first two weeks PI, rectal temperature measurements of both animals were below 39.2°C. Neither steer developed detectable adverse clinical signs or evidence of systemic disease, including respiratory, urogenital, or gastrointestinal disease at any time during the two-week PI period. Infectious BoHV-4 was co-cultured from PBLs on day seven PI from steer 2 and on day 17 PI from steer 1, and was verified by BoHV-4-specific PCR. BoHV-4 was not recoverable from PBLs on any other date. Both calves developed anti-BoHV-4 serologic responses within approximately nine weeks PI, and maintained detectable levels by indirect ELISA for the remainder of the study, with the exception of one time point at week 44 (Fig 1, panel A). Because of the speculation that BoHV-4 infection and resultant pre-existing immunity to the vector could preclude the development of antibody responses to the vaccine antigen after BoHV-4-vectored immunization, we next decided to immunize these two, BoHV-4-immune calves with recombinant BoHV-4-A-CMV-IgK-gE2gD-TM. In this case, development of an antibody response to BVDV and BoHV-1 following BoHV-4-A-CMV-IgK-gE2gD-TM immunization would indicate that BoHV-4 is an effective vaccine vector, even in the face of pre-existing anti-BoHV-4 immunity.

### Immunization of BoHV-4 immune-cattle with BoHV-4-A-CMV-IgK-gE2gD-TM using intranasal nebulization

At the onset of this experiment, the two BoHV-4-immune steers had pre-existing viral neutralizing (VN) BVDV antibody titers, but were negative for BVDV by PCR and E^rns^ antigen testing. Since BVDV is endemic in our region, these data indicate that titers were due to either previous, resolved BVDV infection or lingering maternal antibody from colostrum acquired shortly after birth. Despite this, we chose to include these cattle because successful vaccination would elicit an increase in BVDV VN antibody titers due to an anamnestic response to E2 and the development of anti-BoHV-1 antibody responses would indicate successful vaccination. Indeed, within three weeks of the first BoHV-4-A-CMV-IgK-gE2gD-TM immunization, BVDV VN titers increased logarithmically in both of these calves (Fig 1, panel B). In addition, both steers developed detectable antibody titers to BoHV-1 on indirect ELISA (Fig 1, panel C), but neither developed detectable VN responses to BoHV-1 post immunization (Fig 1, panel D). Because detection of recrudescent BoHV-4 by viral isolation (VI) following dexamethasone suppression of cattle has been described [32], VI was attempted in these cattle after dexamethasone immunosuppression, and was unsuccessful in both steers. However, following dexamethasone suppression 77 weeks after the onset of the experiment, both steers maintained VN antibody responses to BVDV (Fig 1, panel B). The magnitude of BoHV-1 seropositivity increased dramatically based on indirect ELISA (Fig 1, panel C), and both steers developed and maintained VN titers to BoHV-1 within two weeks of dexamethasone administration that remained for the duration of the study (Fig 1, panel C).

### Immunization of BoHV-4-naïve cattle with BoHV-4-A-CMV-IgK-gE2gD-TM using intranasal nebulization

This experiment additionally used two, age-matched BoHV-4 seronegative steer calves, steers 3 and 4, to further evaluate the vaccination serology kinetics. Within twelve weeks after the first immunization, at week 36 after the onset of the experiment, both steers developed detectable BoHV-4 titers (Fig 1, panel A). Steer 4 remained seropositive until week 39. At that point, anti-BoHV-4 antibody titers declined below the level of detection until the animals underwent dexamethasone suppression during week 77, at which point, both steers regained and maintained BoHV-4 seropositivity until the end of the study (Fig 1, panel A). Both calves developed BVDV VN titers within seven weeks after the first immunization, at week 31, and those VN anti-BVDV titers were maintained for the remainder of the experiment (Fig 1, panel B).

At the onset of this experiment, steer 3 had pre-existing antibody titers to BoHV-1 based on the indirect ELISA results (Fig 1, panel C). This positivity is attributed to residual maternal antibody due to the fact that BoHV-1 was not detected via PCR or VI from the peripheral blood or nasal swab specimens at any time point, even following dexamethasone suppression, which reliably causes detectable recrudescence of latent BoHV-1 in the peripheral blood and nasal secretions [33–35]. BoHV-1 ELISA titers increased dramatically in both animals by two weeks after immunization, at week 26 (Fig 1, panel C). Steer 3, which had preexisting BoHV-1 titers developed transient BoHV-1 VN titers detectable on weeks 28 and 31, which declined to undetectable levels until after dexamethasone suppression. Both steers developed VN antibody responses to BoHV-1 within two weeks of dexamethasone suppression (Fig 1, panel D). Viral isolation after immunization and dexamethasone immunosuppression was unsuccessful in both steers.

## Discussion

The results of this study indicate that nebulization of BoHV-4-A-CMV-IgK-gE2gD-TM is capable of eliciting and maintaining BoHV-4-vectored antigen-specific immunity following immunization, including in the presence of pre-existing BoHV-4 immunity. Thus, BoHV-4 is a viable and attractive vaccine delivery platform for use in cattle. Livestock production demands have increased steadily for decades, and are projected to continue to trend upwards on a global scale [36, 37]. As production demands increase, efficient methods of disease prevention in bovine herd health management are necessary. The provision of long-term, or even lifelong, immunity with a single dose of vaccine would be an obvious advantage for many types of cattle operations, including large-scale production systems and small-holder agropastoralists. Additionally, the development of polyvalent recombinant vaccines to immunize against multiple pathogens using a single vaccination would similarly increase the level of efficiency for large-scale vaccination strategies. Given the ability of BoHV-4 to persistently infect cattle and the ability to accommodate large amounts of foreign genetic material, as evidenced by the inclusion of multiple antigens in this study, makes the development and use of BoHV-4 appealing as a vaccine vector for use in cattle.

The decision to pursue the intranasal/intrapulmonary route was based on natural gammaherpesvirus pathogenesis, which establishes infection in mucosal epithelial cells and subsequently undergoes persistent infection within monocytes and macrophages. Previous studies have shown successful infection via nebulization of cattle with the gammaherpesvirus ovine herpesvirus-2 [38]. However, prior studies using recombinant BoHV-4 as a vaccine vector utilized subcutaneous [16] or intravenous (IV) [11, 12] administration. Specifically, immunization of rabbits using a BoHV-4-A-CMV-IgK-gE2gD-TM construct was performed via IV injection. Since the efficacy of BoHV-4 infection and immunization after intranasal/intrapulmonary administration had not been previously verified, we used nebulization to infect two calves with WT BoHV-4, and successfully established infection in both steers. Due to the timing of experimental infection and lack of any associated clinical evidence of disease, the transient fever detected in both steers on day seven likely reflects primary viremia associated with acute BoHV-4 infection. Similar findings have been reported in other experimental BoHV-4 infections of cattle inoculated via the intramammary, intradermal, and IV methods [6]. Detection of virus in the blood of one of these steers on this day further corroborates this hypothesis, and BoHV-4 was detected via PBL co-culture and verified by PCR in the other steer on day 17 PI. Although the dogma of herpesvirus infections dictates that all infections are persistent, PCR-based diagnostic tests and direct viral culture of blood can be difficult, especially after approximately three weeks post- experimental inoculation of BoHV-4 [5]. Consistent with these results, we were unable to culture virus from PBLs in either steer after the second week following infection with WT BoHV-4. Both steers developed a variably detectable anti-BoHV-4 antibody response over the course of approximately one year post-immunization. Despite successful WT BoHV-4 infection and subsequent anti-BoHV-4 immune response development in these calves, both animals developed immune responses to recombinant vaccine antigens after subsequent superinfection with BoHV-4-A-CMV-IgK-gE2gD-TM. Thus, previous exposure to natural BoHV-4 infection and the presence of preexisting anti-BoHV-4 antibodies should not be considered a deterrent or disadvantage to immunization with recombinant BoHV-4, consistent with what has been described with other herpesvirus vectors [39, 40]. Although these two steers had pre-existing VN antibody titers to BVDV at the onset of the study, they tested negative for the BVDV E^rns^ antigen and were PCR negative for BVDV at all time points throughout the study, thereby ruling out BVDV reinfection as the cause of the logarithmically increased anamnestic BVDV VN titer observed following BoHV-4-A-CMV-IgK-gE2gD-TM immunization. That data, coupled with the development of anti-BoHV-1 antibody titers following BoHV-4-A-CMV-IgK-gE2gD-TM immunization, supports the use of BoHV-4 as a vaccine vector, even in animals previously infected and with active immune responses to BoHV-4.

In contrast to the anti-BoHV-4 serologic response observed following WT BoHV-4 inoculation, anti-BoHV-4 antibody levels were not sustained above cut-off levels in the two steers immunized with the recombinant virus after 15 weeks post-immunization, on week 39 of the study. This may have resulted from the lower immunization dose of recombinant BoHV-4-A-CMV-IgK-gE2gD-TM compared to WT BoHV-4, or a decreased ability of recombinant virus to establish infection or replicate *in vivo*. Previous data indicate that various BoHV-4 recombinants, including the BoHV-4-A-CMV-IgK-gE2gD-TM construct, maintain essentially equal growth curves *in vitro* [12]. However, since the starting titer of the two purified virus aliquots differed, a direct comparison could not be established in this study. *In vivo* infection, growth, and latency characteristics of WT BoHV-4 or recombinant BoHV-4-A-CMV-IgK-gE2gD-TM have not been fully characterized in any species, including the natural host.

Despite the less robust anti-BoHV-4 antibody response observed following immunization with BoHV-4-A-CMV-IgK-gE2gD-TM, both steers developed VN anti-BVDV and non-neutralizing antibody titers to BoHV-1. Steer 3, immunized with only the recombinant BoHV-4-A-CMV-IgK-gE2gD-TM construct, was seropositive for BoHV-1 at the onset of the study. This is suspected to reflect maternal antibody interference since this animal never developed a detectable BoHV-1 viremia and had no evidence of BoHV-1 infection based on PCR testing and post-dexamethasone VI. Thus, the increase in anti-BoHV-4 antibody titers, coupled with the transient detection of VN anti-BoHV-1 antibodies after immunization with BoHV-4-A-CMV-IgK-gE2gD-TM, indicate that this animal developed an antigen-specific anamnestic response to the BoHV-1 gD vaccine antigen.

Following dexamethasone induced immune suppression, an increase in anti-BoHV-4 titer was observed in all four cattle used in this study. This result, in combination with sustained VN anti-BVDV titers and the development of VN anti-BoHV-1 titers in all cattle without clinical and molecular evidence of reactivated latent BoHV-1 infection, is consistent with persistence of the recombinant virus and resultant recrudescence and boosting of the immune response to the included immunogens. In this case, the magnitude of the VN anti-BVDV and anti-BoHV-1 VN antibody responses is consistent with clinical protection from disease dictated by the Code of Federal Regulations (9 CFR § 113.215 and 113.216, respectively) [25]. The stability of the VN anti-BVDV titers in the face of dexamethasone suppression is consistent with previous evidence that indicates that dexamethasone suppression does not reduce the VN activity in cattle that have preexisting BVDV VN antibodies [41].

The precipitous increase in anti-BoHV-1 antibodies detectable by ELISA, and the development of BoHV-1 neutralizing antibodies in all four cattle following dexamethasone suppression is interesting. One possibility considered to explain this logarithmic seroconversion is that these animals were previously exposed to BoHV-1 and were in a state of clinical latency without detectable evidence of BoHV-1 infection. Dexamethasone suppression is considered the method of choice to reactivate latent alphaherpesviruses, specifically BoHV-1, and viremia is consistently detectable by PCR from peripheral blood [42]. In our study, dexamethasone suppression did not yield any evidence of BoHV-1 infection via VI and PCR testing of the peripheral blood, nasal secretions, or postmortem tissue samples, thus making this an unlikely scenario. Post-dexamethasone gD subunit studies are lacking in bovine vaccinology research, however, antibody responses tend to increase in dexamethasone suppressed, latently BoHV-1-infected animals [42]. Another possibility considered is that BoHV-4-A-CMV-IgK-gE2gD-TM incited vector-neutralizing antibody responses. Although this possibility of internal neutralization could suggest impedance of the vector to elicit antibody responses, it also suggests that potential *in vivo* recrudescence of live BoHV-1 was neutralized, based on the inability to detect BoHV-1 infection, and would also indicate success of the vaccine. Regardless, an increase in the seropositivity post-dexamethasone suppression indicates a source of persistent antigen exposure. In the absence of any evidence of infection with natural BoHV-1, the most likely antigen source is the recombinant BoHV-4-A-CMV-IgK-gE2gD-TM.

In previous studies, successful co-culture of infectious BoHV-4 from PBLs is sporadic during the acute infection and undetectable in later stages of infection [29]. This was consistent with our results following nebulization of WT BoHV-4. However, another previous study described the detection of recrudescent BoHV-4 by culture of the peripheral blood following dexamethasone suppression of cattle in the absence of detectable clinical signs [32]. Thus, VI was attempted following dexamethasone-induced suppression. No infectious virus was detected in circulation via co-culture of PBLs, nasal secretions, or in post-mortem tissue samples including the lung, liver, kidney, spleen, tracheobronchial lymph node, trigeminal nerve ganglia, or bone marrow. It should be noted that in these previous studies, VI after dexamethasone administration from the peripheral blood required no less than 10^8^ cells, and even then detection via PCR evaluation is sporadic [32]. Additionally, dexamethasone administration occurred within two months of the initial experimental infection in these studies, as opposed to almost one year following BoHV-4-A-CMV-IgK-gE2gD-TM immunization and 1.5 years after initial WT BoHV-4 infection in our studies. In our studies, approximately 10^3^ PBLs were collected and tested from each steer for each time point, thereby markedly decreasing the chance of successful isolation. The effect of timing of dexamethasone administration in relation to initial infection has not been explored in regards to detectability of BoHV-4, but the possibility that this detrimentally affected the ability to culture virus was considered in this study. To the author’s knowledge, co-culture of BoHV-4 from post-mortem tissue samples has not been described on dexamethasone treated cattle.

As previously stated, the decision to immunize via the intranasal route was made to most accurately mimic the natural pathogenesis of gammaherpesviral infection, resulting in optimization of infection efficiency. Intranasal immunization has other fortuitous functional and logistical advantages. First, intranasal delivery of an antigen has been shown to produce potent systemic protective immunity, due at least in part to the fact that the upper respiratory mucosa is rich in lymphoid tissue and functional antigen presenting dendritic cells [43]. Therefore, the development of a protective immune response is expected and has been widely utilized in veterinary vaccine development [44, 45]. Additionally, the noninvasive nature and ease of administration of intranasal vaccines facilitates widespread vaccine implementation [46, 47]. Lastly, IN vaccines are needleless and therefore decrease the risk of iatrogenic blood-borne pathogen transmission. These properties of intranasal vaccines tremendously increase the field-use potential for use in livestock production management.

Our data supports the use of recombinant BoHV-4 as a vaccine vector for cattle. Most significantly, these findings indicate that BoHV-4-vectored vaccines are able to elicit protective immunity to included antigens following immunization of cattle; robust immune responses to BoHV-4-vectored antigens are elicited following immunization with recombinant BoHV-4, even in the face of pre-existing BoHV-4 immunity; recombinant BoHV-4 persists in cattle following immunization, and recrudescence of the virus boosts the immune response to BoHV-4-vectored antigens; and the recombinant BoHV-4 vaccine can be delivered via the intranasal route. These findings emphasize the substantial potential of recombinant BoHV-4-vectored bovine vaccines, and provide rationale for further studies in BoHV-4 vectored vaccine development.
